# A Review of the Progress and Challenges of Developing a Vaccine for COVID-19

**DOI:** 10.3389/fimmu.2020.585354

**Published:** 2020-10-14

**Authors:** Omna Sharma, Ali A. Sultan, Hong Ding, Chris R. Triggle

**Affiliations:** ^1^Weill Cornell Medicine-Qatar, Doha, Qatar; ^2^Department of Microbiology and Immunology, Weill Cornell Medicine-Qatar, Cornell University, Doha, Qatar; ^3^Departments of Medical Education and Pharmacology, Weill Cornell Medicine-Qatar, Education City, Doha, Qatar

**Keywords:** SARS-CoV-2, COVID-19 pandemic, vaccine development, DNA vaccine, RNA vaccine, non-replication viral vector vaccine, inactivated virus particle vaccine, neutralizing antibodies

## Abstract

A novel coronavirus, which has been designated as severe acute respiratory syndrome coronavirus 2 (SARS-CoV-2), was first detected in December 2019 in Wuhan China and causes the highly infectious disease referred to as COVID-19. COVID-19 has now spread worldwide to become a global pandemic affecting over 24 million people as of August 26th, 2020 and claimed the life of more than 800,000 people worldwide. COVID-19 is asymptomatic for some individuals and for others it can cause symptoms ranging from flu-like to acute respiratory distress syndrome (ARDS), pneumonia and death. Although it is anticipated that an effective vaccine will be available to protect against COVID-19, at present the world is relying on social distancing and hygiene measures and repurposed drugs. There is a worldwide effort to develop an effective vaccine against SARS-CoV-2 and, as of late August 2020, there are 30 vaccines in clinical trials with over 200 in various stages of development. This review will focus on the eight vaccine candidates that entered Phase 1 clinical trials in mid-May, including AstraZeneca/Oxford's AZD1222, Moderna's mRNA-1273 and Sinovac's CoronaVac vaccines, which are currently in advanced stages of vaccine development. In addition to reviewing the different stages of vaccine development, vaccine platforms and vaccine candidates, this review also discusses the biological and immunological basis required of a SARS-CoV-2 vaccine, the importance of a collaborative international effort, the ethical implications of vaccine development, the efficacy needed for an immunogenic vaccine, vaccine coverage, the potential limitations and challenges of vaccine development. Although the demand for a vaccine far surpasses the production capacity, it will be beneficial to have a limited number of vaccines available for the more vulnerable population by the end of 2020 and for the rest of the global population by the end of 2021.

## Introduction

In December 2019, an outbreak of the coronavirus disease 2019 (COVID-19) emerged and was first identified in Wuhan, China and then quickly spread to now become a global pandemic affecting, as of August 26th 2020, more than 24 million people worldwide with the US comprising almost 6 million cases. COVID-19 has been attributed to the novel severe acute respiratory syndrome coronavirus-2 (SARS-CoV-2) and the illness has caused a spectrum of clinical manifestations ranging from asymptomatic, minor flu-like symptoms to acute respiratory distress syndrome (ARDS), pneumonia and death. It is anticipated that the COVID-19 pandemic can be controlled using social distancing, masks, new antiviral drugs and an effective vaccine. Although developing herd immunity through acquiring natural immunity via infections is possible, the death toll and consequences as such would be devastating (1). This was seen in Sweden where authorities presumed that by infecting up to 60% of the population, herd immunity would be sufficient to protect the more vulnerable population (2). However, this failed and the deaths per million population attributed to COVID-19 in Sweden is at least 5 times that of Germany (2). Hence, developing an effective vaccine is crucial and considered the only practical way to establishing herd immunity.

Researchers around the world are aggressively working around the clock to develop a vaccine against COVID-19. As of late August 2020, there are more than 200 vaccine candidates in various stages of development. While there are 30 vaccines currently in clinical trials, this review will focus on the 8 vaccines that entered Phase 1 clinical trials in mid-May including AstraZeneca/Oxford's AZD1222 and Moderna's mRNA-1273 vaccines. Although the production capacity may not be able to meet the global demand for vaccines in the very near future, it would be beneficial to have a limited number of vaccines available for emergency use and the more vulnerable population as soon as possible with the ultimate aim of distributing vaccines globally to the rest of the population by the end of 2021.

In order to develop a safe and effective vaccine, it is critical that pre-clinical and clinical trials are done with vigilance to avoid severe adverse effects (3). Furthermore, cooperation between international organizations such as the World Health Organization (WHO), Coalition for Epidemic Preparedness Innovations (CEPI), Gavi alliance, Accelerating COVID-19 Therapeutic Interventions and Vaccines (ACTIV) and Bill and Melinda Gates Foundation (BMGF) amongst others is essential to ensure adequate funding for vaccines and a collaborative response to the COVID-19 pandemic (3). This review summarizes the biology and immune response demonstrated from previous coronavirus infections and SARS-CoV-2, the various platforms being utilized for COVID-19 vaccine candidates, describes an outline of the process of traditional vaccine development, examines and analyses the progress of 8 different vaccine candidates and outlines the challenges associated with vaccine production in a pandemic. In addition, the question of whether mutations in the spike protein might affect the efficacy of a vaccine is addressed as also are potential problems that may arise by fast-tracking vaccine production. Vaccine development has typically taken up to 15 years, but with fast tracking it is hoped to reduce this to 1.5 years or less thus potentially raising concerns over public acceptance as well as concerns regarding challenges from anti-vaxxers.

### SARS-CoV-2

Coronaviruses are enveloped, positive-sense single-stranded RNA viruses with a helical nucleocapsid. They belong to the *Coronaviridae* family in the order *Nidovirales*, subfamily *Orthocoronaviridae* and are divided into four genera namely alpha, beta, delta, and gamma coronavirus (4). Severe acute respiratory syndrome coronavirus-2 (SARS CoV-2) is a beta-coronavirus belonging to the same group as severe acute respiratory syndrome coronavirus (SARS-CoV) and Middle East Respiratory Syndrome coronavirus (MERS-CoV). Although it is unclear as to how the virus was first transmitted to humans, its origins can be traced to bats, with bats also the original source for other coronavirus infections in humans (5, 6) and also Ebola (7). A study looked at cross-sectional and case-series studies primarily from China and upon analysis, the studies have shown that the mean age of patients diagnosed with COVID-19 was 52 years old with 55.9% of patients being male (8). The most common clinical manifestations included cough, fever, myalgia or fatigue with more than half of patients developing dyspnea (8, 9). Fever was seen more commonly in adults than in children (8). The most prevalent laboratory results included elevated C-reactive protein (CRP), elevated lactate dehydrogenase (LDH), lymphopenia and decreased albumin (8). Higher prothrombin times and D-dimer levels were noted for those admitted to intensive care units (ICU) (9). 36.8% of patients presented with comorbidities - the most common being hypertension, cardiovascular disease and diabetes (8).

The structure of SARS-CoV-2 involves a major trimeric envelope glycoprotein called the S-protein, which is expressed on the surface of the virus and is also the main target for vaccines as it binds to host cells. The S-protein is made of two main subunits namely S1 that controls receptor binding and S2, which governs membrane fusion (10). The S protein also undergoes a significant conformational change from a pre-fusion state to a post-fusion state, achieved by pulling and fusing the cell and viral membranes together (11). The S protein in coronaviruses is quite diverse as supported by the fact that the S proteins for SARS CoV and MERS CoV only share 44% of the genetic sequence (10). The differences in the S protein are primarily attributed mainly to the S1 subunit, which is composed of an N-terminal domain (NTD) and a receptor-binding domain (RBD). The diversity of RBD between SARS-CoV and MERS-CoV is attributed to different host cell entry receptors for the two coronaviruses namely angiotensin converting enzyme 2 (ACE2) for SARS-CoV and also for SARS-CoV-2 while dipeptidyl peptidase 4 (DPP4) is the receptor for MERS-CoV (10, 12). Since SARS-CoV and SARS-CoV-2 share the same entry receptor, monoclonal antibodies against SARS-CoV RBD were tested for cross-reactivity to SARS-CoV-2 RBD and results showed that no binding was detected to SARS CoV-2 RBD despite the similarity in RBD sequences (12). In terms of the severity and clinical consequences of the infection, SARS-CoV was more lethal and aggressive but SARS-CoV-2 is highly contagious and spreads more readily (13). Furthermore, another caveat with SARS-CoV-2 is that in some individuals the symptoms are hidden or the individual is asymptomatic, meaning that potentially an infected person unknowingly infects multiple people (13). Epidemiological studies conducted in China have estimated that the so-called reproduction number (*R*_0_) used as a measure of how many others an infected person can potentially infect is 3 (14). The highly infectious nature of SARS-CoV-2 has led to millions of cases worldwide and reinforced the global need for an effective vaccine to stop the spread of disease and reduce the number of deaths.

### Immune Response to SARS-CoV-2 and Previous Coronavirus Infections

A strong and potent immune response is essential to clear the SARS-CoV-2 infection from the human body. A study published in the journal *Cell* showed that infected individuals had a strong T cell response to the virus, which may help them recover from the virus (15, 16). The results showed that all of the patients carried helper T cells that recognized the spike protein on SARS-CoV-2 (15, 16). These patients also had helper T cells against some of the other proteins on SARS-CoV-2. These data indicate that T cells do play a role in eliminating SARS-CoV-2. Helper T cells stimulate B cells to further release antibodies and helper T cells also stimulate cytotoxic T cells. Cytotoxic T cells were demonstrated in 70% of patients (15, 16). Interestingly, 34% of uninfected individuals in the same study were shown to have helper T cells that could respond to a SARS-CoV-2 infection (15, 16). Further analysis of the blood samples collected from 2015 to 2018 revealed that these helper T cells could have been triggered from a previous coronavirus infection since there is some similarity in S proteins between the different coronaviruses (15, 16). It is also worth mentioning that the debate as to the origins of SARS-CoV-2 continues with several reports that the virus may have been circulating much earlier than November-December 2019 with emerging, but to be confirmed, evidence of its presence in sewage samples as early as March 2019 as reported by Spanish researchers from Barcelona (17). Previous exposure to SARS-CoV-2, or a close relative, may explain the low number of cases and deaths reported in countries such as Vietnam. In another study published in *Nature* the T cell response to the nucleocapsid protein (NP) of SARS-CoV-2 and also the memory T cell response to the NP protein of SARS-CoV were investigated. The results showed that patients who recovered from COVID-19 demonstrated both CD4^+^ and CD8^+^ T cells against the NP protein (18). They also illustrated that individuals who have recovered from a SARS-CoV infection still possess T cells specific to SARS-CoV, particularly against the NP protein, and additionally, these T cells have demonstrated the ability to cross react with the SARS-CoV-2 NP protein (18). Analysis of the presence of SARS-CoV-2 T cells in uninfected individuals was also conducted and the results showed that SARS-CoV-2 specific reactive T cells were detected but to a lesser extent for the NP protein and interestingly to a greater extent for other proteins. These findings support the theory that the T cell immune response can be stimulated following exposure to other beta coronaviruses (18). Thus, lasting immunity from a previous coronavirus infection could help protect against SARS-CoV-2 and also raises the possibility that long-lasting T cell immunity will persist in COVID-19 recovered patients (15, 16, 18).

Studies on antibody responses to SARS-CoV-2 infections are ongoing with most studies illustrating that those who recover have antibodies to the virus (19). However, the level of SARS-CoV-2 specific neutralizing antibodies (NAbs) has shown to be varied between different groups of populations, which supports the theory that T cell response also plays an important role in clearing the SARS-CoV-2 infection (19). Elderly patients were shown to be more likely to develop high levels of SARS-CoV-2 specific NAbs compared to younger patients, suggesting a strong innate immune response but whether the high levels of NAbs protect such patients from progression into the critical phases of COVID-19 requires further evaluation (19).

Plasma cells and memory B cells that emerge in response to a primary infection are involved in long-term protection against a reinfection. There is intense interest surrounding the memory B cell response from SARS-CoV-2 and previous coronavirus infections. Results from studies designed to analyze antibodies from COVID-19 infection showed that IgG antibody titers rose in the first 3 weeks following symptom onset (20, 21). Although the IgG titer levels dipped in the second month following symptom onset, the level was still above the threshold and was detectable in the serum, indicating the possibility of protection against a reinfection but more research is needed to better profile the timeline of the IgG response. Examination of the immune response following a SARS-CoV infection indicated that specific IgG response declined within the first 2 years of infection and was detectable in all patients in the first 16 months but was noted to be almost undetectable in about 11.8% of patients in the 24th month (21, 22). Likewise, for the SARS-CoV specific NAb response, NAb levels were detectable up to 2 years after infection but the levels started to decline in the 16th month of follow up (21, 22). Moreover, the study also showed that the rate of decline in NAb levels was faster in men than women but, again, more research needs to be done to determine the accuracy and cellular basis for this observation. Answers to these questions as well as the influence of the age of the patient are needed to better understand the predicted effectiveness of a vaccine.

Several studies and reports have emphasized the NAb responses to different COVID-19 vaccines. Although such knowledge is important, it is also equally crucial to consider T cell responses as these are known to be more durable and provide long lasting immunity. Some reasons as to why the T cell response has not been previously emphasized include that it is more challenging to test for T cell response in trial participants especially in a larger population (23). It is important to ensure that a vaccine is eliciting not only a higher number of NAbs but also a good T cell response to ensure long lasting and effective immunity against SARS-CoV-2 (23).

## COVID-19 Vaccine Development

**Figure 1:**
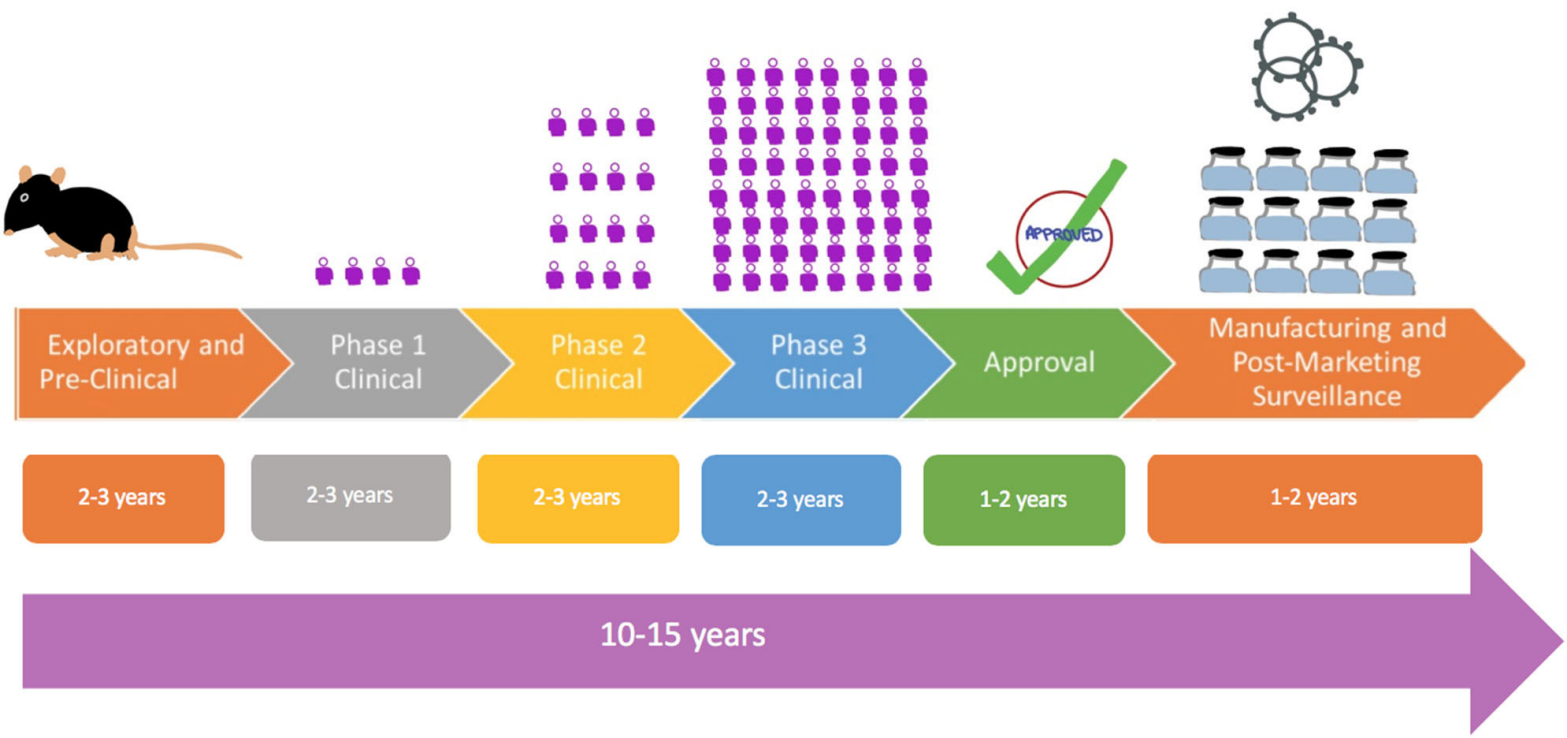
Flowchart showing traditional process of vaccine development from exploratory, pre-clinical studies to Phase 1 studies in a comparatively few control volunteers as depicted by the figure to larger Phase 2 and Phase 3 studies. The 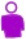 symbol is a representation of the number of human subjects in trials.

To date the development of a new vaccine has been a long process that typically takes anywhere from 10 to 15 years (24), as shown by Figure 1. The fastest that a vaccine has been developed and approved for use is for mumps, which took approximately 5 years. Hence, it is clearly a challenge to develop a vaccine against COVID-19 in a span of 12–24 months. The first phase of vaccine development is an **exploratory stage** involving basic laboratory bench research and computational modeling to identify natural or synthetic antigens that can be used as a vaccine candidate, which might help prevent or treat a disease. The second stage comprises **pre-clinical studies**, which involve cell-culture or tissue-culture systems and trials on an animal model to assess the safety of the candidate vaccine and its immunogenicity, or ability to provoke an immune response. Once safety, immunogenicity and efficacy are demonstrated on animals, progress is made to human clinical trials which test for safety and immunogenicity in small groups then large groups over 3 phases, as outlined below.

**Phase 1 - Safety:** This is the first stage where the vaccine is administered to humans. The vaccine is given to a small number of healthy and immunocompetent individuals to primarily test for safety, appropriate dose and to check for immune response, as a secondary effect.

**Phase 2 - Expanded Safety:** The vaccine is given to hundreds of people split into different groups by demographics (example: elderly vs. young). These again test primarily for safety, appropriate dosage, and interval between doses and check for immune response, as a secondary effect. This phase serves to confirm the vaccine is safe and immunogenic and also determines the appropriate dose to be used in Phase 3 trials.

**Phase 3 - Efficacy:** This is a large-scale trial where the vaccine is given to thousands of people to evaluate efficacy. Vaccine efficacy (VE) is defined as the percentage by which the rate of disease incidence is reduced in vaccinated groups as compared to placebo (25). Incidence of disease at the time of Phase 3 trials impacts the sample size. In the case of a low incidence of disease in the population, a large sample size will be needed to adequately determine vaccine efficacy.

Once the human clinical trials are completed, and the safety and the clinical efficacy have been determined, then the vaccine will move to:

- **Review and Approval:** Normally, regulatory bodies, such as Food and Drug Administration (FDA) of the USA, or European Medicines Agency in EU, must review the results from clinical trials and decide if the vaccine is fit to be approved. As this process can take anywhere from 1 to 2 years, vaccines may be approved for emergency use in a pandemic.
- **Manufacturing and Post-Marketing Surveillance:** This is done after the vaccine is marketed for public use and monitored for general effectiveness within the population. They also record adverse effects that might be experienced after the vaccine is adopted for widespread use.

**Figure 2:**
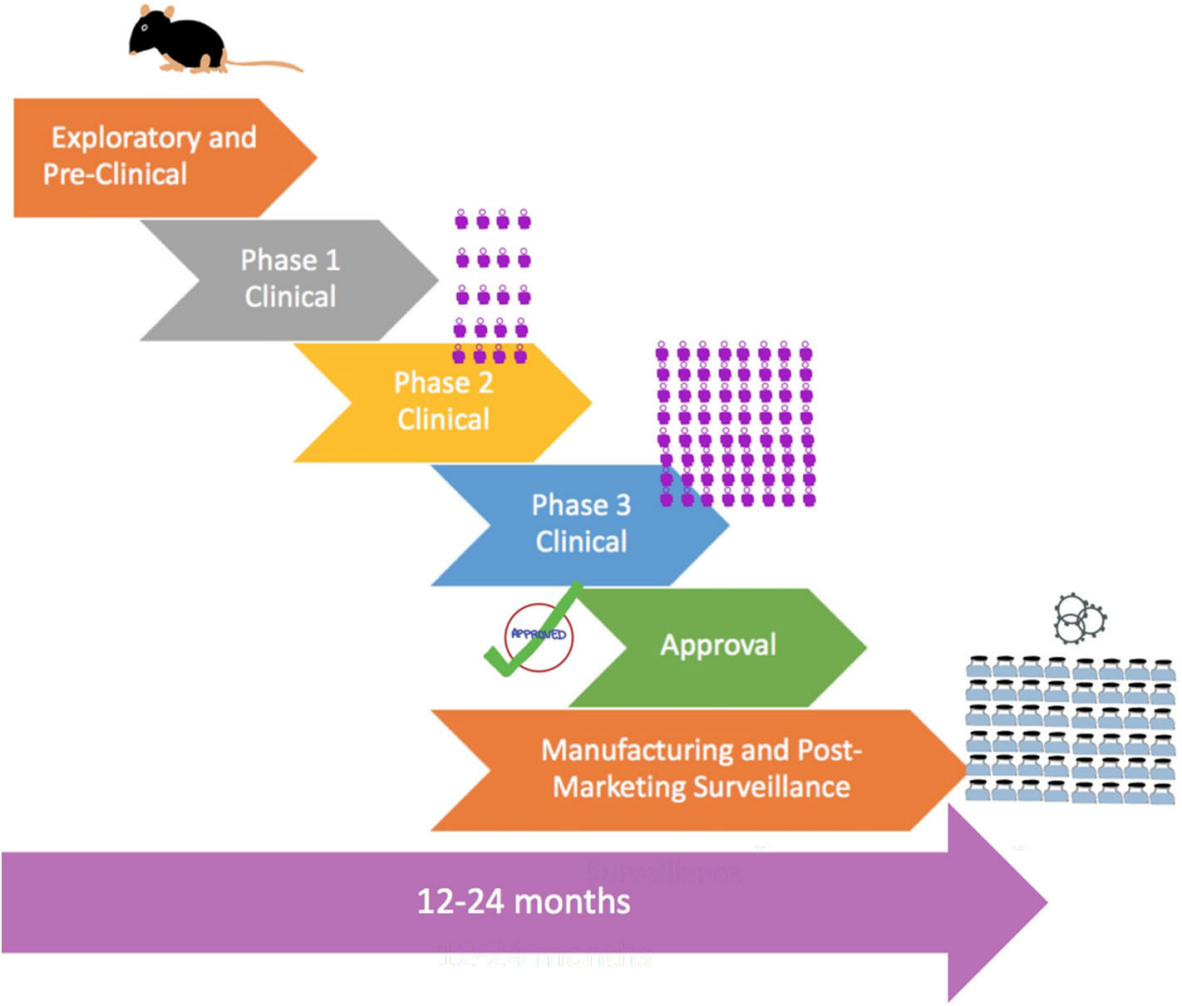
Flowchart showing accelerated process of vaccine development in a pandemic with combined phases, pre-approval, and rapid large-scale manufacturing. The 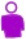 symbol is a representation of the number of human subjects in trials.

Given the upheaval caused by the COVID-19 pandemic and the urgent need for an effective vaccine globally, vaccine development can be accelerated by combining phases, as shown by Figure 2. An example would be combining Phases 1 and 2 to test for safety in hundreds of people directly. Vaccines also do not go through the full approval process and may instead be approved for emergency use for quicker release for use by the most vulnerable groups. Of significance is that 5 vaccines have been selected by the White House for its *Operation Warp Speed* program to accelerate vaccine development and have them available by the end of 2020 for emergency use and have billions of doses by 2021.

### Various Platforms for COVID-19 Vaccine Development

**Figure 3:**
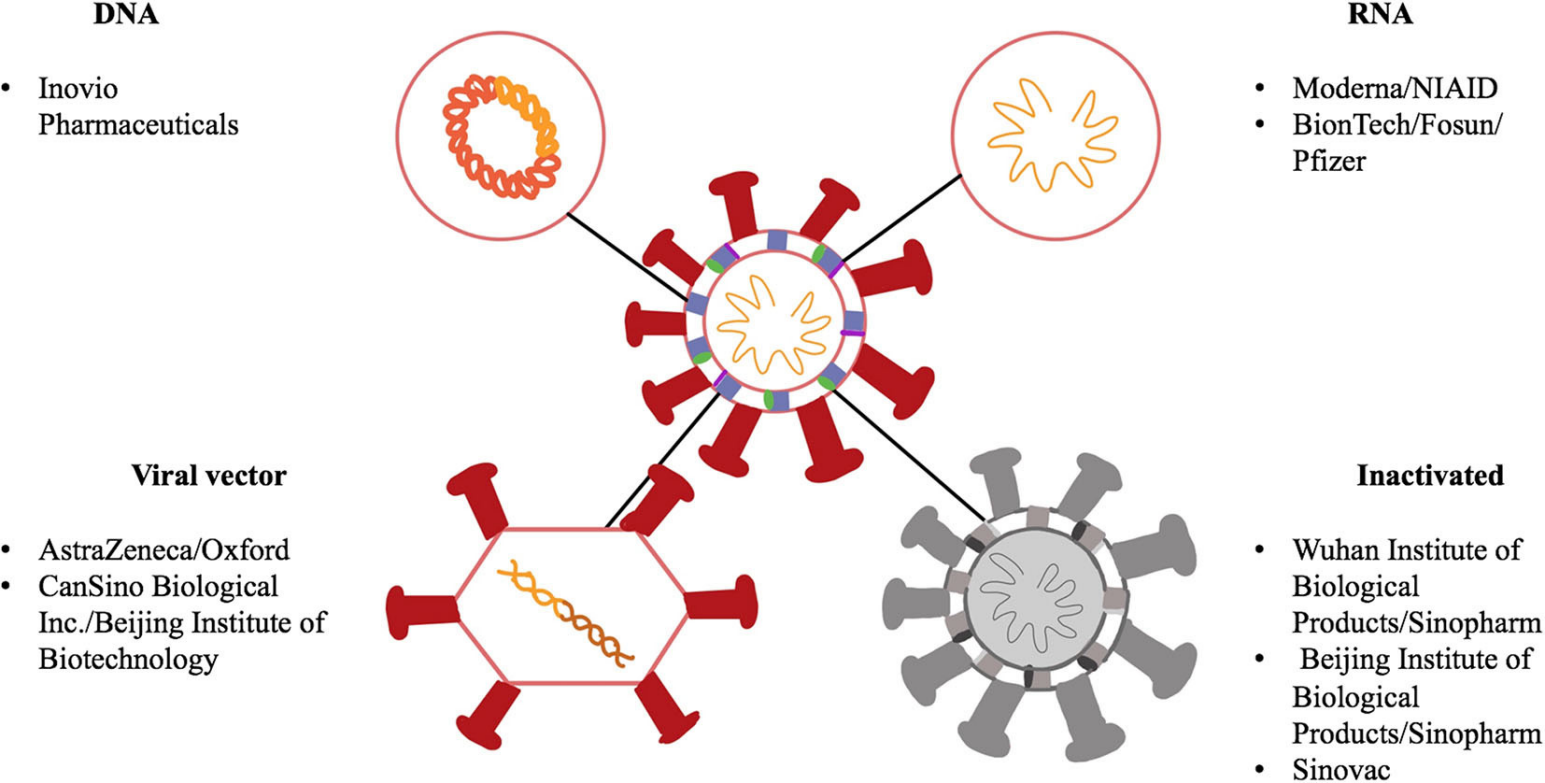
Schematic showing a representation of SARS-CoV-2 along with different components of the virus as potential vaccine targets. SARS-CoV-2 is a single stranded RNA virus, has a lipid bilayer and consists of a spike S protein along with membrane and envelope proteins. DNA and RNA-based vaccines are made from the viral sequence of the virus. Viral vector vaccines utilize another virus, for example an adenovirus, and incorporate genetic material from SARS-CoV-2 into its genome. Inactivated vaccines involve SARS-CoV-2 that has been killed using physical or chemical means.

There are various platforms being looked at for the development of COVID-19 vaccines. These include RNA, DNA, non-replicating viral vectors and inactivated vaccines. These platforms are illustrated in Figure 3. While RNA and DNA based vaccines have not been developed and licensed for human use in the past, these two platforms do provide an advantage in a pandemic situation. Since both of these platforms do not require bio reactor culture techniques as would be needed, for instance, for an inactivated vaccine, they can be made more rapidly in the laboratory and are based on the genetic sequence of the virus and allows for the development process to be fast-tracked in the event of a pandemic (26). They are also able to generate a robust immune response, which provides an added benefit. In contrast, non-replicating viral vector vaccines can be manufactured on a large scale and have shown to be safe and effective immunologically as seen with an Ebola vaccine candidate (27). On the other hand, vaccines based on inactivated virus technology have been licensed previously but they do not generate as strong of an immune response unless used alongside, as an example, an aluminum adjuvant. Therefore, given the urgent need and demand of a vaccine in this global pandemic, it is not surprising there are several DNA, RNA as well as non-replicating vector vaccines in clinical trials even though there have been no previously licensed vaccines produced based on the DNA or RNA platforms. Table 1 showcases 8 different vaccine candidates along with some characteristics of the different vaccine platforms.

**Table 1:** Platforms and candidates of vaccines being used for COVID-19 along with data on their doses, speed^2^, immune response, advantages and disadvantages.

**Platform**	**Candidates in clinical trials and phase^a^**	**Type of candidate vaccine**	**Target antigen**	**Single/** **multiple dose**	**Speed^b^**	**Immune response**	**Advantages**	**Disadvantages**
DNA	Inovio Pharmaceuticals - phase 1/2	DNA plasmid vaccine with electroporation	Spike protein	Multiple	Fast	Both humoral and cellular	-Electroporation generates a robust immune response -Made using genetic sequence and does not need to be cultured	-Although deemed to be safe, electroporation can be complicated and potentially problematic. -No DNA based vaccine has been previously produced
RNA	Moderna/NIAID - phase 3	Lipid nanoparticle [LNP]-encapsulated mRNA	Spike protein	Multiple	Fast	Both humoral and cellular	-Made using genetic sequence and does not need to be cultured	-LNP is temperature sensitive -Ability to manufacture large scale unknown -No RNA based vaccine has been produced before
	BioNTech/Fosun Pharma/Pfizer - phase 3	3 LNP-mRNAs	Spike protein					
Non-replicating viral vector	AstraZeneca/University of Oxford - phase 3	AZD1222	Spike protein	Single	Medium	Both humoral and cellular	-Can be manufactured large scale -Safe and effective immunologically as shown with Ebola	-Pre-existing immunity could hamper clinical use and reduce immune response
	CanSino Biological Inc./Beijing Institute of Biotechnology - phase 2	Adenovirus type 5 vector	Spike protein					
Inactivated	Wuhan Institute of Biological Products/Sinopharm - phase 3	Inactivated	Whole virus	Multiple	Medium	Mostly humoral	-Pathogen is killed and hence, no risk of reversion	-Risk of vaccine-enhanced disease -Usually produce a weak immune response
	Beijing Institute of Biological Products/Sinopharm - phase 3		Whole virus					
	Sinovac - phase 3	Inactivated + aluminum adjuvant	Whole virus			Mostly humoral - aluminum adjuvant enhances response more robust		

a *Phase for vaccine development is as of August 20th, 2020*.

b *Speed refers to how quickly a vaccine candidate is able to progress through different stages of vaccine development considering the nature of the platform being utilized. Adapted from the WHO COVID-19 Vaccine R&D Landscape (28) and others (29–31). Candidates listed in red are part of Operation Warp Speed to accelerate vaccine development and production*.

#### DNA-Based Vaccines

##### Inovio

Inovio Pharmaceuticals is an American company based in Plymouth Meeting Pennsylvania, USA, that specializes in manufacturing DNA-based drugs and vaccines and has a COVID-19 vaccine, INO-4800, currently in Phase 1/2 clinical trials. The clinical trial is split into two parts - A and B. For part A, Inovio initially recruited 40 healthy adults between the ages 19 and 50 in South Korea to test the vaccine for safety and immune response (32). The vaccine is injected intradermal followed by electroporation to ensure uptake into cells. In their studies the participants were split into two groups for either a low (1 mg) or a high dose (2 mg) trial and were administered two doses 4 weeks apart (32). According to their press release, 3 participants (1 from the low dose and 2 from the high dose group) were dropped from the trials since they tested positive for COVID-19 and one participant from the high dose group was dropped for undisclosed reasons that was claimed to be not related to safety or immunogenicity (33).

Based on interim data from a press release, 34 out of 36 (94%) patients enrolled in the trial demonstrated an immune response at week 6 (33). Participants were contacted to check for adverse events periodically and interim data show that at week 8, 10 out of 36 (28%) individuals reported Grade 1 adverse events, which were mild fever and reactions that did not interfere with daily routine (33). They plan to recruit additional participants and expand the age group by incorporating 51–64-year-olds for the Part B component of their Phase 1/2 trials (32). The number of participants in its initial phase 1/2 trials is too few to make quick assumptions about the vaccine despite its supposedly increased immune response and mild adverse events. Since Inovio has not published any data from its clinical trials, the specific details from the safety and immunogenicity of the vaccine are yet to be seen and its slow progress in clinical trials leaves a lot of questions unanswered.

#### RNA-Based Vaccines

##### Moderna/NIAID

Moderna is another American company based in Cambridge, Massachusetts that is developing an mRNA-based vaccine, mRNA-1273. The mRNA vaccine codes for the spike protein such that when the vaccine is injected into the body, the immune cells processing the mRNA and the manufactured protein will be subsequently marked for destruction (34). Moderna's vaccine is included in the *Operation Warp Speed* initiative to accelerate vaccine production. It is currently in Phase 3.

Moderna released interim data from its preclinical trials in the journal *Nature* (35). It is worthwhile to note that this data was released after Moderna had published preliminary data on its Phase 1 trials. They tested their vaccine in mice by immunizing them with either the 0.01, 0.1, or 1 μg dose of the vaccine intramuscularly (35). Results showed that a high pseudo virus NAb response was seen with the 1 μg dose. Additionally, a high pseudo virus NAb response was also seen in mice expressing the mutated form of the spike protein, D614G, which is now beginning to be seen in cases worldwide (35). Furthermore, the 1 μg dose illustrated a robust cytotoxic T cell response along with a balanced Th1/Th2 response (35). This is important because a dominant Th2 response is linked to vaccine-associated enhanced respiratory disease (VAERD). It was also noted that no increased pathology was observed in the mice upon administration of the vaccine at a dose of 1 μg (35). The level of NAb response in a 1 μg dose in mice was stated to be comparable to a 100 μg dose in humans, thus supporting the selection of a 100 μg dose for large scale efficacy trials (35).

Its Phase 1 trials recruited 45 healthy participants of ages 18–55 years old (36). Participants were split equally into 3 groups to account for 3 different doses (25, 100, and 250 μg) (36) Two doses were administered intramuscularly 28 days apart. Two participants (1 in the 25 μg group and 1 in the 250 μg group) who were suspected of exposure to COVID-19, but later tested negative, missed their second dose. Based on a published preliminary report, interim results show that no serious adverse events were reported but one participant experienced transient urticaria, a hives rash, after the first 25 μg dose and was withdrawn from obtaining the second dose (34). There was no fever reported post the first dose but some participants in the 100 (6 out of 15; 40%) and 250 μg (8 out of 14; 57%) groups reported fever after the second dose (36). Local adverse events were primarily Grade 1 and Grade 2, with pain at the injection site being a commonly reported event (36). In addition, participants reported other systemic and local adverse effects including myalgia, headaches, fatigue and chills after both doses. Three patients in the 250 μg group (21%) reported severe systemic adverse effects following the second dose (36).

A specific antibody response was apparent depending on the dose administered and peaked at day 15 after the first dose (36). NAbs were detected in only less than half of the participants following the first vaccination but were detected in all participants following the second vaccination which infers the need for a two-dose vaccine regimen (36). A lower response was noted in the 25 μg group and high responses were noted in the other two dose groups (36). CD4^+^ T cell responses were detected with the 25 and 100 μg doses with an additional low CD8^+^ T cell response shown following a second 100 μg dose (36). Moderna is yet to release results from a second group consisting of older participants aged 55 and above and since older individuals have a reduced immune response, it will be important to see the dosage used and if any side-effects result from the possibly higher dose (37).

Moderna's Phase 2a trial involved 600 healthy participants recruited from the ages 18 and above to test for safety and observe adverse reactions and to also check for immunogenicity (38). This was a randomized, double blind trial which split the participants based on age and dose into 8 groups - 4 were taking 50 and 100 μg of the vaccine and the other 4 were taking 50 and 100 μg of saline (placebo) (38). A Phase 3 trial was initiated at the end of July 2020 and is designed to test for efficacy by evaluating the 100 μg dose of the vaccine (35, 36, 39) administered on days 1 and 29. This is a randomized trial incorporating quadruple blinding (39). Moderna aims to recruit 30,000 participants' aged 18 and above in the United States divided into either the vaccine group or placebo group. It has set a broad inclusion criteria, which includes those who have pre-existing conditions provided such conditions are stable and do not require changes in their therapy in the 3 months prior to enrollment (39).

Based on the Phase 1 interim results, the two-dose regimen Moderna has chosen certainly showed an immune response in a greater number of individuals but also reported side effects, although mostly mild to moderate, have also increased following the second dose (36). It will be interesting to see the outcome in the older Phase 1 group and the larger Phase 2 and 3 clinical trials.

##### BioNTech/Fosun/Pfizer

BioNTech, a German company, together with Pfizer, an American company, are developing another mRNA-based vaccine, which encodes the SARS-CoV-2 RBD domain. This vaccine candidate, named BNT162, incorporates modified mRNA and also includes a T4 fibritin-derived trimerization domain to enhance immune response (40). Currently in Phase 3 trials, BioNTech/Pfizer is a candidate that is part of *Operation Warp Speed*. For their phase 1/2 trials in the USA, 45 healthy volunteers were recruited between the ages 18 and 55, split into groups of 12 for different doses (10, 30, and 100 μg) and a group of 9 participants receiving a placebo (40). Two doses for the 10 and 30 μg were administered intramuscularly 20 days apart; the group with 100 μg dosage did not receive a second dose (40).

Based on interim data, participants showed increased IgG levels, which heightened 7 days after the second dose (28-day mark) and remained elevated until 14 days after the second dose (35-day mark) (40). For those that received a 100 μg dose, IgG levels peaked at 21 days after the first dose and did not increase thereafter (40). For NAb titers, elevated levels were observed 21 days after the first dose and 7 days after the second dose (28-day mark) (40). Since the 100 μg group did not receive a second booster dose, no data about immunogenicity is available for that group. Furthermore, results showed that there were no significant differences in immune response between the 30 and 100 μg groups after the first dose (40). These data argue for the 10 and 30 μg doses as better candidates and thus, are more likely to proceed through future trials (40).

For the BNT162 vaccine dose-dependent Grade 1 to Grade 2 systemic or local reactions were noted. Pain at the injection site was a common event and was predominantly mild or moderate with the exception of one severe event in the 100 μg group (40). Commonly occurring systemic events included fatigue, headache, chills, muscle and joint aches. These symptoms increased in severity based on the dose and although particularly severe after the second dose did resolve within a day (40). Some patients reported fever following the first and second doses but these resolved within 1 day (40). No Grade 4 adverse events were reported. However, a few participants complained of Grade 3 pyrexia and sleep disturbance. Laboratory values did not change much for most individuals but a few were noted to have decreased lymphocyte and neutrophil count which then returned to normal 6–8 days post-vaccination (40).

#### Non-replicating Viral Vector Vaccines

##### AstraZeneca/University of Oxford

The University of Oxford has formed a partnership with the British pharmaceutical company AstraZeneca to develop a non-replicating chimpanzee viral vector vaccine, formerly known as ChAdOx1 and now designated AZD1222. Currently leading the clinical trials race, AZD1222 is in Phase 3 and is also part of the *Operation Warp Speed* initiative. Preclinical trials in pig models demonstrated a high antibody response (41). A Phase 1/2 trial was completed and the results were reported in the journal *Lancet*. They conducted a randomized, single-blinded trial on 1077 healthy participants, aged between 18 and 55 and recruited in the UK (42, 43). These participants received either the AZD1222 vaccine at a dose of 5 × 10^10^ vaccine particles (*n* = 543) or a placebo licensed meningococcal vaccine MenACWY (*n* = 534) (43). A group of 10 participants in the AZD1222 group received a second booster dose of the vaccine 28 days following the first dose (43). The dose for the AZD1222 vaccine was selected based on the Oxford group's prior experience with developing a similar type of ChAdOx1 vaccine for MERS (43).

Participants were also divided based on paracetamol (acetaminophen) prophylaxis as this was used to monitor a reduction in adverse events. Fifty six out of 543 participants in the AZD1222 group and 57 out 534 participants in the placebo MenACWY vaccine group were given paracetamol (43). Results showed that local and systemic adverse events were noted to a lower degree in the paracetamol group as compared to the group with no prophylaxis (43). This finding was also replicated in the placebo groups. In those who received paracetamol, fewer patients reported pain, tenderness, fatigue and headache compared to the non-paracetamol prophylactic group (43). Other less frequently observed events in the group given paracetamol include myalgia, chills and fever (43). These events were reported to be mild to moderate in range and were highest in severity a day after vaccination. However, it is interesting to note that neutropenia was observed in 46% (25 out of 54) of the participants in the AZD1222 group compared to 7% (3 out of 44) of the control MenACWY group (43).

By day 28, specific antibodies peaked in the AZD1222 vaccine group and these levels remained elevated until day 56 (43). Additionally, by day 56, a much higher specific antibody response was noted for the 10 participants who received a booster shot (43). Immune response was not affected by the prophylactic use of paracetamol. A high NAb response was seen in 91% of participants across different assays after the first dose. All participants of the booster dose group had a high NAb response thus supporting the need for a two-dose regimen to increase the NAb response (43). T cell response, observed in all participants, peaked at day 14 and remained elevated through day 56 (43). However, participants in the booster group did not observe an increase in T cell response following the second dose (43).

This Phase 1/2 trial study had some limitations including the very few number (*n* = 10) of chosen participants for the booster group (43). Since the benefit of a booster dose is apparent on increasing specific and NAb response, it is important that more participants are recruited for the booster group to confirm the finding in large-scale trials and rule out any risk of antibody-dependent enhancement (ADE) of COVID-19.

In addition, a Phase 1/2 trial is ongoing on 2,000 volunteers with or without HIV in South Africa aged 18–65 to check for safety and immune response (44). Participants were split into groups based on varying doses of the vaccine or placebo (44). A phase IIb/III trial involved 12,330 healthy UK volunteers and included those above 5 years of age (45). Participants were split into groups based on age and included cohorts of extreme demographics (5–12 years old and above 70 years old) who are at greater risk from COVID-19 (38). Furthermore, participants were split into groups to either receive the AZD1222 vaccine or a licensed meningococcal MenACWY vaccine as a control (45).

The phase III trial that is now in progress involves over 30,000 volunteers in the United States, Brazil, South Africa and India (43, 46, 47). As per their clinical trial protocol, 2,000 volunteers will be recruited in Brazil where they will receive one shot of 5 × 10^10^ vaccine particles of the AZD1222 vaccine or 0.5 ml of meningococcal MenACWY vaccine as a placebo (46). Furthermore, volunteers will also be asked to take paracetamol for 1 day after the vaccination (46). Additionally, 30,000 volunteers are being recruited at various sites across the United States where volunteers will either receive 2 doses of 5 × 10^10^ vaccine particles of the AZD1222 vaccine separated by 4 weeks or a saline placebo (47). Further details and results for these clinical trials are yet to be made available.

##### CanSino Biological inc./Beijing Institute of Biotechnology

CanSino's Ad5-nCoV vaccine is another non-replicating viral vector vaccine utilizing the Ad5 adenovirus to insert the SARS-CoV-2 gene into the human body. In the past CanSino has successfully been involved in the production of an Ebola vaccine. Published data from the its Phase 1 trials in the journal *Lancet* showcased that no adverse reactions were observed within 28 days post-vaccination for the Ad5-nCoV vaccine (48). CanSino conducted its safety trials on 108 healthy adults in Wuhan between the ages 18 and 60 who were split equally into one of three dose groups (5 × 10^10^ viral particles or 1 × 10^11^ viral particles or 1.5 × 10^11^ viral particles) to test for effects of dose-escalation (48). The most common reported reactions were pain at the site of injection in addition to fever, muscle aches, headaches and fatigue (48). Ten individuals experienced these symptoms at the Grade 3 level with 6 being in the high dose group and accounting for 17% of the high dose group (48). Additionally, some patients reported hyperglycemia, increased levels of total bilirubin and 5 alanine aminotransferase but these were not considered to be clinically significant (48). It was reported that NAb titer levels increased 14 days post-vaccination and peaked 28 days post-vaccination. The T cell response was heightened 14 days post-vaccination (48). Since this vaccine utilizes a human adenovirus, the presence of pre-existing immunity against adenoviruses was considered and results showed that pre-existing immunity to adenovirus showcased diminished NAb levels and T cell response (48).

On July 20th, CanSino published its Phase 2 trial results in the *Lancet* (49). They conducted a randomized, double-blinded clinical trial on 508 healthy, HIV-negative participants above 18 years of age (49). The participants were given one intramuscular injection of the vaccine, either 1 × 10^11^ viral particles (*n* = 253) or 5 × 10^10^ viral particles (*n* = 129), or a placebo (*n* = 126) (49). It was shown that by day 28, specific antibodies peaked to a much higher degree for the 1 × 10^11^ group at 656.5 geometric mean antibody titers (GMT) and 571.0 GMT for the 5 × 10^10^ group with high seroconversion rates of 96 and 97%, respectively (49). By day 28, NAbs also peaked for both groups with the 1 × 10^11^ group achieving a higher response with a GMT of 19.5 and the 5 × 10^10^ group receiving a GMT of 18.3 (49). However, only 59% of individuals in the 1 × 10^11^ group and 47% participants in the 5 × 10^10^ group demonstrated NAb response, thus raising questions about the effectiveness of the immune response in this vaccine (49). Furthermore, it was noted that 52% of the participants had a high level of pre-existing immunity to adenoviruses (49). As such, those with a low level of pre-existing adenoviral immunity reported up to 2 to 3 times higher immune response against SARS-CoV-2. It was also noted that the older group consisting of participants above 55 years of age demonstrated a lower antibody response, particularly the NAbs but both antibody titers were still higher relative to the placebo (49).

Both vaccine groups reported mild to moderate adverse events such as fatigue, fever, headache and pain at the injection site (49). Up to 24% of the 1 × 10^11^ vaccine group, a percentage significantly higher than the 5 × 10^10^ vaccine group and placebo, reported a severe Grade 3 adverse event including fever which self-resolved (49). Based on these results, CanSino has indicated that the vaccine dose with 5 × 10^10^ viral particles will be used in forthcoming Phase 3 trials (49).

On June 25th the China's Central Military Commission approved the use of Ad5-nCoV by the military for a period of 1 year –arguably the equivalent of a Phase III trial (50). Additionally, CanSino will be conducting Phase 3 trials in Saudi Arabia but data about the logistics of the trial have not yet been made available (51).

##### Comparing Oxford/AstraZeneca's and CanSino's vaccine candidates

Both Oxford/Astrazeneca and CanSino utilize adenovirus as a vector for their COVID-19 vaccine. Adenoviruses are common and can cause a variety of illnesses in humans ranging from a cold to conjunctivitis (52). When comparing the NAb response between the two adenoviral vector-based vaccine candidates, it was shown that while Oxford/AstraZeneca's AZD1222 has demonstrated a high NAb level in 91% of individuals following the first dose, and in all individuals following a booster dose, only 59% of individuals in CanSino's vaccine demonstrated a NAb (43, 49, 52). This indicates that a good proportion of participants did not develop an effective immune response due to the presence of pre-existing immunity against human adenoviruses. Oxford/AstraZeneca were able to prevent this outcome by utilizing a genetically modified chimpanzee-derived adenovirus against which humans do not have pre-existing immunity (43, 52). However, CanSino plans to offers its vaccine at a low cost which, combined with its moderate efficacy, may prove advantageous for some countries (52).

#### Inactivated Vaccines

##### Wuhan Institute of Biological Products/Beijing Institute of Biological Products/Sinopharm

Sinopharm is developing two inactivated vaccines in collaboration with Wuhan Institute of Biological Products and Beijing Institute of Biological Products. Both vaccine candidates are currently in Phase 3 trials. Wuhan Institute of Biological Products released interim results for its double blind and randomized Phase 1 and 2 clinical trials in the journal *JAMA* (53). In the phase 1 trial, 96 participants aged between 18 and 59 were recruited and equally assigned to one of three dose groups (2.5 μg or 5 μg or 10 μg) or an aluminum adjuvant placebo group (53). These participants received three intramuscular shots at days 0, 28 and 56 (53). On day 7, adverse reactions were reported by 20.8% (5 out of 24) in the low dose group, 16.7% (4 out of 24) in the medium dose group, 25% (6 out of 24) in the high dose group and 12.5% (3 out of 24) participants in the aluminum adjuvant placebo group (53). Commonly reported adverse reactions were pain at injection site and fever which were mild and self-resolved (53). 14 days after the third vaccination (day 70), a high NAb response was observed with seroconversion being observed in all participants in the low and high dose groups, 95.8% (23 out of 24) participants in the medium dose group (53). A specific antibody response was also generated to high levels in this phase 1 trial and seroconversion was observed in all participants (53).

In the phase 2 trial, 224 participants aged between 18 and 59 were recruited and equally assigned to one of two dual-dose programs - days 0 and 14 or days 0 and 21 (53). In each schedule, 84 were assigned to the medium dose (5 μg) vaccine group and 28 were assigned to an aluminum adjuvant placebo group (53). It is to be noted that for the immunogenicity component of Phase 2 trials, only the first half of the participants were analyzed within each group. For example, for the day 0 and 14 schedule, 42 were included in the 5 μg dose group and 14 in the placebo group for the immunogenicity component but for safety analysis, all 84 in the 5 μg group and all 28 in the placebo group were considered (53). For the 0- and 14-day schedule, 6% (5 out of 84) in the 5 μg group and 14.3% (4 out of 28) participants in the placebo experienced adverse reactions. For the 0- and 28-day schedule, 19% (16 out of 84) in the 5 μg group and 17.9% (5 out of 28) in the placebo group had adverse reactions (53). As in the phase 1 trial, fever and pain at injection site were commonly reported mild events that resolved on their own (53). A high NAb response was seen in both schedules with a 97.6% (41 out of 42) seroconversion noted for both (53). Additionally, for the specific antibody response, a much higher response was shown with the 0- and 21-day schedule than the 0- and 14-day schedule (53). Seroconversion was also relatively low in the 0- and 14-day schedule with 85.7% (36 out of 42) compared to 100% for the 0- and 21-day schedule (53). This supports the fact that a higher gap between doses is correlated with a higher immune response in this vaccine.

T cell responses were not measured in either trial and hence it is not known if this vaccine can cause VAERD. This potential problem needs to be investigated in large-scale efficacy trials to illustrate both humoral and cellular immune responses. Additionally, the report on phase 2 trials did not analyze all participants for the immunogenicity component of the trial and perhaps, this could create a false sense of security when interpreting the elevated humoral immune response results.

Biological Institute of Biological Products released results pertaining to their pre-clinical trials. A high NAb response was achieved at all doses (2 μg or 4 μg or 8 μg) along with an aluminum adjuvant across different animal species including rats, mice, macaques and cynomolgus monkeys (54). Furthermore, neither high nor low doses of the vaccine were associated with ADE of the disease in the study with macaques (54). It was noted that two doses of the 2 μg dose conferred a highly effective immune response without causing ADE or other immunopathological effects and therefore considered for clinical trials (54). The company also conducted a randomized, double blind, parallel phase 1/2 trial that recruited 1,120 healthy participants aged between 18 and 59 (55). Participants were split into dose and age-dependent groups for either receiving the inactivated vaccine or a placebo (55). Adverse reactions were noted periodically and humoral and cellular immune responses were assessed (55). Based on interim data from a press release, it was noted that a high antibody response was observed with no significant adverse events, but further details are awaited, as no published data has been made available (56). For both of their Phase 3 trials, Sinopharm is looking to conduct them in the United Arab Emirates due to too few active COVID-19 cases in China (57) with plans to recruit up to 15,000 volunteers.

##### Sinovac

Sinovac is currently developing an inactivated + aluminum adjuvant vaccine, CoronaVac, which is currently in Phase 3 trials. Its Phase 3 trials are being conducted in Brazil and Indonesia due to fewer active cases in China (58, 59). Data published from pre-clinical trials in mice and macaque models showed that sufficient specific IgG response and NAb titer levels were achieved (60). The mice were injected with either 1.5 μg or 3 μg or 6 μg doses of the vaccine along with an alum adjuvant or a saline placebo (60). No ADE was noted in the macaque monkeys that were vaccinated. Furthermore, vaccinated macaques were challenged with SARS-CoV-2 and they were noted to be protected from the virus with decreased viral loads unlike the control group (60). It is important to ensure safety especially in the case of inactivated vaccines and a dose of 6 μg of CoronaVac was found to be protective and had no changes in mental status or appetite and no other side effects were noted in the macaque monkey (60).

A press release for their Phase 1 study mentioned that they recruited 143 healthy participants aged between 18 and 59 for a randomized and double-blinded trial but no results pertaining to the phase 1 study have been made available (61). Phase 2 trials involved 600 participants between the ages 18 and 59 in a randomized, double-blinded trial (62). The participants were split into two dual-dose programs - either the 0- and 14- day or 0- and 28- day schedule (62). Within each schedule, 120 participants were administered the 3 μg dose, 120 participants were administered the 6 μg dose and 60 participants were given a placebo (62). Local adverse events such as pain and swelling were mild to moderate with pain being the most common reported event in both schedules (62). Sixty one participants out of 300 (20.3%) in the 0- and 14-day schedule and 31 out of 300 (10.3%) participants in the 0- and 28-day schedule complained of pain at the injection site post-vaccination (62). These adverse events resolved within 3 days (62). No severe Grade 3 adverse events were reported (62).

NAb responses were high for both 3 and 6 μg doses in both schedules (62). 28 days after the second dose, those in the 0- and 14-day schedule had stable NAb levels but for the 0- and 28-day schedule, NAb levels increased considerably (62). A similar pattern was observed for specific antibodies as well (62). It was also noted that NAb levels diminished with increased age thus suggesting an increased dosage requirement for the elderly (62). T cell immunity was not analyzed in this report and further data is required to provide a complete picture of the immune response generated by the CoronaVac vaccine. Knowing about the T cell response of the vaccine is also necessary to rule out the risk of ADE, as it is known to be associated with the use of inactivated vaccines. Although pre-clinical studies showed no immunopathological findings, it remains to be seen if a similar finding is replicated in human clinical trials.

Sinovac plans to assess the 3 μg dose in the 0-, 14- day and 0-, 28-day schedules in large-scale efficacy trials in Brazil and Indonesia (58, 59, 62). In Brazil, Sinovac is assessing its vaccine over a 0- and 14- day schedule and plans to recruit 8,874 healthcare workers that are above the age of 18 (58). Their large-scale efficacy trials include the elderly above 60 years of age and it will be very interesting to see the outcome achieved in the elderly population (58).

## International Collaboration

In order to ensure that the threat of COVID-19 is eliminated, it is critical that a coordinated and cooperative approach is taken which includes the collaboration between several international organizations to ensure that a process to ensure that sufficient financing and fair distribution of the vaccine supply is available. GAVI, the Vaccine Alliance is one such organization, which is a global public-private partnership to ensure that individuals from developing countries, particularly children, have access to immunizations (63). It is also part of the recent Global Vaccine Summit, which allocated funding for COVID-19 vaccine development and also to healthcare systems of GAVI-eligible countries and adequate supply for developing countries. In addition, Bill and Melinda Gates Foundation (BMGF) have allocated $250 million toward development of vaccines and for supporting the health care systems of Sub-Saharan Africa and other developing countries (64). Coalition for Epidemic Preparedness Innovations (CEPI) is a foundation that is involved in financing vaccine development and has launched COVID-19 Vaccine Global Access Facility (COVAX) in order allow for equal access of COVID-19 vaccines for countries (65). Lastly, the WHO is very much involved in all aspects of COVID-19 pandemic including ensuring vital equipment and personal protective equipment (PPE) such as masks and medical gowns for health care workers, research for COVID-19 vaccines, providing accurate information pertaining to COVID-19 and coordinating with countries for a response to COVID-19 amongst others (66). The WHO is also documenting data from vaccine candidates in its Draft Landscape of COVID-19 vaccine being updated periodically (28). Additionally, cooperation from individual countries is also equally important in the fight against COVID-19.

## Ethical Concerns Surrounding Vaccine Development

Given the urgent need of a vaccine, vaccine development and production are being fast-tracked to hopefully make a safe and effective vaccine available by the end of this year for the more vulnerable group of the population. However, perhaps linked to the upcoming political election in the USA in November of 2020, the White House initiated the *Operation Warp Speed* program to develop vaccines at an accelerated speed. Moderna's mRNA vaccine and AstraZeneca/University of Oxford's AZD1222 vaccine are part of this program and, given their progress thus far, it is possible that their vaccines will be available by the end of 2020. However, production managers of vaccine candidates have reported feeling pressured to develop a vaccine in a span of months when the traditional process on average takes well-over 10 years (67). The Trump administration has also allocated billions of dollars in funding for these vaccines. However, fast tracking and rushing vaccines could prove to be detrimental as this might result in producing a vaccine that is not optimally effective and may only provide immunity, or incomplete immunity, to some vaccinated individuals (68). Although it is assumed that through the scrutiny of the academic and scientific community a vaccine will not be released for use to the public before all appropriate safety and efficacy tests have been performed, it is important to consider the recent small-scale human trials of the Russian vaccine, Sputnik V, even though in the absence of published data it's use appears to have bypassed safety trials (69). Russia has also begun manufacturing its vaccine with plans to administer it in the Philippines and potentially other countries (70). To date no data pertaining to the safety and immunogenicity of the Russian vaccine has been published although a report from Reuters on August 21st indicated that a Phase 3 study to include more than 40,000 people was to be initiated and, in addition, data on the Sputnik V vaccine was to be published soon (69, 71). To maintain public trust in vaccines it is important that full transparency in all aspects of vaccine development is available.

Another concern is that when clinical trials are being done on comparatively small groups of people, and fast-tracked from one phase to the next there is a risk of masking side-effects that would have been detected if the vaccine was tested in larger populations. Furthermore, it is important to consider whether there was an appropriate demographic consideration in the design of the clinical trials - different races, varying age groups and those with comorbidities - if not, this may lead to unforeseen outcomes upon vaccinating these individuals when the vaccine is released for public use. Hence, given an accelerated timeline, post marketing surveillance becomes of greater importance, as this will provide the necessary vigilance as to the effectiveness of the vaccine along with recording adverse effects once it is released for public use. However, it is also important to note that vaccines developed following the traditional timeline can also be at risk for unforeseen adverse effects. This was noted in Philippines when the French dengue vaccine, Dengvaxia, was administered and caused unexpected complications resulting in over 500 deaths, particularly in previously uninfected school-going children (72).

Several vaccine candidates including Moderna and AstraZeneca/Oxford are planning or entering Phase 3 trials, which require studying effectiveness in approximately 30,000 people. Since several countries are now starting to see a drop in the number of infections, there is a concern as to how will these clinical trials will acquire a sufficient number of people to conduct the studies. Taking advantage of the difference in transmission rates around the world, the Phase 3 trials for some vaccine candidates including AstraZeneca/Oxford's AZD1222 will be conducted in areas with higher COVID-19 infections such as Brazil, USA, India and South Africa thus overcoming, in the case of AZD122, the decrease in disease prevalence in the UK (43, 46, 47). Another way to address this concern includes conducting human challenge trials (HCT). In HCTs volunteers are deliberately infected with the virus in order to monitor their response to the vaccine. Although HCTs have been used relatively safely in the past as they help accelerate vaccine development, the risk-benefit analysis does not align as perfectly in the case of COVID-19. Thus, as of late August 2020 there are still a lot of unknowns about the variable pathogenesis of COVID-19 in the population and there is no proven licensed treatment available and hence there exists a very significant potential risk of the development of severe disease in the volunteers for an HCT (73). Although a risk-benefit analysis can be conducted on an individual basis and assess an individual's health status before inclusion in an HCT, nonetheless, this will likely greatly limit the generation of data being available for the elderly and those with comorbidities.

In addition, given the demand for vaccines, several countries including the United States and Europe have indicated that the vaccines will be initially provided to their own citizens. However, questions are being raised regarding the ethics of fair allocation. Although AstraZeneca has announced collaboration with an Indian institute for supply of adequate doses to low- and middle-income countries, it remains to be seen as to how the allocation of the vaccine when it is approved and becomes available (74). It is also important to prioritize certain groups of people for vaccine allocation including health care workers, the immunocompromised, those with comorbidities, the elderly and those with lower socioeconomic status to ensure distributive justice (75).

## Efficacy of the Vaccine, Potential Limitations, and Coverage

With the development of vaccines and clinical trials underway, questions arise as to how much efficacy is needed for the vaccine to be immunogenic. While more research is still needed, preliminary research studies have shown that while an efficacy ≥70% is needed to eliminate the infection, a prophylactic vaccine with an efficacy <70% will still have a major impact and may contribute to eliminating the virus, provided appropriate social distancing measures (76). Vaccines with an efficacy below 70% may also contribute to reducing the duration of infection in those infected with the virus (76).

Given what we know about SARS CoV-2 thus far, the diversity observed between the pandemic sequences of SARS CoV-2 is low (77). However, the widespread presence of the pandemic can cause natural selection to act upon certain mutations (76). There has been a D614G mutation on the spike S protein - a G to A base change from the original Wuhan strain - found primarily in Europe and has been shown to have increased transmissibility and a higher viral load but more research is needed to determine its impact on clinical outcomes (77). Although the D614G mutation is located on the spike S protein, it is not in the RBD but rather in between the individual spike protomers to provide stability through hydrogen bonding (78). This means that while it may have an impact on the infectivity of the virus, it should not drastically affect the effectiveness of vaccines and consequently the NAbs produced against the RBD (78).

Another concern is the phenomenon of ADE of COVID-19 disease and it should be taken into consideration when developing vaccines against SARS-CoV-2. ADE has been observed with other coronaviruses including MERS-CoV and SARS-CoV (78). ADE occurs when antibodies bind to the virus and the resulting antibody-virus complex facilitates viral entry by host macrophages instead of neutralizing the virus (11, 79). Inactivated vaccines particularly pose a risk as they can cause VAERD as has been seen in the past with measles and respiratory syncytial virus (RSV) in humans and with SARS-CoV in animal models (11). VAERD is due to the presence of increased numbers of antibodies that do not neutralize the virus when a high viral load is present (11). This consequently results in immune complex deposition and can lead to severe respiratory disease (11). While ADE is more of a concern for inactivated vaccines, these phenomena should be kept in mind for other COVID-19 vaccine platforms as well (80). Given the urgent global need for a COVID-19 vaccine, being overly pre-cautious should not restrict the release of an otherwise well-tolerated, safe and immunogenic vaccine (80).

Several vaccine candidates are being developed from small-scale companies, such as Moderna, that until COVID-19 are not well-known and have not previously produced an effective vaccine but, nonetheless, have their COVID-19 vaccine in clinical trials. In contrast, vaccines from well-known companies such as GSK and Pfizer are currently in Phase 1 and Phase 1/2 clinical stage, respectively (81). Having vaccine candidates from several developers spread out over various stages in testing and trials is a positive outlook for the future since hopefully this will result in the availability of several effective vaccines that could then meet global needs. At the very least having multiple vaccine candidates at various stages of development and testing boosts confidence that should one vaccine fail in the clinical trials there are other alternative vaccines in development.

The threat of anti-vaxxers is an ever-present danger and already evident as vaccine opponents are refuting statements by experts pertaining to vaccines. Anti-vaxxers and their false theories and influence paved the way for the worst measles outbreak in the United States in 2019 and experts fear similar consequences for COVID-19. Several surveys in May found that between 14 and 23% of Americans are not willing to be vaccinated with 22% claiming they are unsure (82). Another poll showed that only 49% of Americans were willing to take a COVID-19 vaccine when one becomes available (83). These numbers shed light on the growing anti-vaccine sentiment in the community, which can prove to be dangerous and result in high numbers of deaths. The anti-vaccine sentiment is not limited to the US alone. Europe, which witnessed a tripling of measles cases in 2018, Germany and Australia too have a fair share of anti-vaxxer communities. Conceivably even with the worldwide availability of an effective vaccine millions will refuse to accept it. To counter this possibility their has to be a strong and effective scientific and political support demonstrating the safety and efficacy of all approved vaccines.

While experts and scientists are doing their best to convince the population of the benefits of a COVID-19 vaccine, which is apparent by the number of vaccines under clinical trials and the funding being directed toward vaccines to obtain one before 2021, there is a concern about rushing vaccines and producing one with limited effectiveness. Furthermore, if a vaccine is approved for use but subsequently is shown not to be as effective as expected in the population, this could lead to loss of trust in vaccines. In consequence, when an effective vaccine is introduced fewer people may be willing to accept it resulting in a worsening of the pandemic and further reduce the confidence in already approved and effective vaccines for other diseases. Thus, it is important to build trust and effective communication and restore public confidence in the public health system, which includes being transparent and reporting accurate data timely pertaining to vaccines (84).

Although mandating vaccine uptake may be considered as an approach for ensuring herd immunity against COVID-19, certain criteria should be fulfilled before such a policy is put in place (85). One of these include that the Advisory Committee overseeing immunization practices has recommended certain high-risk groups such as the elderly or health-care workers for mandatory vaccines (85). Some other criteria include evidence that COVID-19 is not effectively contained in the region, evidence that information about the vaccine's safety and efficacy is communicated to the public in a transparent manner, presence of adequate supply of the vaccine and evidence that not enough members of high-risk groups are taking the vaccine voluntarily (85). Lastly, should an adverse reaction to a vaccine occur, programs should be in place to ensure that there is compensation provided to these individuals and that a record is kept of adverse events to re-evaluate safety (85). With these criteria, responsible authorities should ensure that an effective and fair policy is in place such that if the need for mandatory vaccine is deemed necessary, public trust in the health care system is not jeopardized.

## Summary of Challenges Associated With Vaccine Production for COVID-19

Over the past 4 months, several companies have been accelerating their vaccine production programs. Vaccine development traditionally takes 10–15 years and to condense this to a period of only 15 months comes with its own drawbacks and challenges.

- Accelerating vaccine development by combining phases involves trials being done on smaller groups. This is a significant concern since when the vaccine is released for public use globally, unknown side-effects may appear in the larger population which were previously not observed within smaller groups. Furthermore, if all demographics (elderly and young) and those with comorbidities are not appropriately considered in the design of the clinical trials, there is a chance that unwarranted side-effects may be observed in those groups when the vaccine is available for public use (86). Post-marketing surveillance would ensure that vaccines are monitored for such side effects in the general population.
- In the past, platforms based on nucleic acids such as DNA and RNA have not resulted in a successful vaccine for human diseases and hence it is yet to be seen how mRNA vaccines will be successfully developed since lipid nanoparticles are temperature-sensitive and this may pose difficulties for scaling up production (29). Furthermore, for DNA vaccines, its reliance on electroporation or an injector delivery device for vaccine administration is a potential issue. Although electroporation is considered to be a safe procedure and is critical to generate an increased immune response, it can complicate vaccine delivery (87).
- Pre-existing immunity to adenoviruses is a concern, particularly for those vaccine candidates utilizing human adenoviruses such as CanSino's Ad5 vaccine, as it may result in a reduced immune response to the vaccine (49, 52). AstraZeneca/Oxford's AZD1222 is another adenoviral vector vaccine candidate but instead of utilizing a human adenovirus in its vaccine, it uses a genetically modified chimpanzee-derived adenovirus (43). This effectively addresses the concern about pre-existing immunity and consequently averts the negative impact on immune response generated to the vaccine (43, 52).
- Rapid large-scale manufacturing of vaccines still remains a challenge with lots of uncertainty to meet the demand of a pandemic.
- There are concerns that political pressure to rush the development and approval processes for a vaccine, which may result in an ineffective vaccine being released for public use. Such a scenario may lead to the public being hesitant from accepting future vaccines (67, 68).
- Although the Global Vaccine Summit has called for an equal allocation of vaccines when a vaccine is released, there is still a concern that some countries will want to secure the vaccine supply for their citizens. A similar concern has been expressed as a result of the recent stockpiling in the USA of the drug, remdesivir, for the treatment of patients with COVID-19 (88).
- Phase 3 trials require over 30,000 volunteers and since these trials are performed during the later stages of development there is a high chance that at that stage there will be fewer cases of COVID-19 and hence, HCTs may be required. Although HCTs have been done in the past, they may pose more risk for COVID-19 given how there is very little known about the pathogenesis and the availability of an effective treatment for COVID-19 (89). As an alternative to HCTs, several vaccine candidates have also utilized the differing transmission rates to their advantage by conducting phase 3 trials in countries with a higher SARS-CoV-2 infection rate to ensure that an adequate number of participants are able to partake in Phase 3 trials.
- Mutations of the virus can result in vaccines having limited effectiveness against it (76, 90).
- There is also risk of vaccine-enhanced disease for inactivated vaccine candidates, notably VAERD that needs to be kept in mind (11, 27). Furthermore, ADE is a known risk for vaccines developed for coronaviruses and a similar concern is being echoed for SARS-CoV-2 and should be kept in mind when developing vaccines against COVID-19 (79). However, given the crucial need for the global availability of a COVID-19 vaccine globally, being concerned and assessing such risks should not prevent the release of otherwise safe and effective vaccines to the public (80).

## Future Outlook and Concluding Thoughts

As of August 2020, many are hopeful that by the end of 2020, or early 2021, an effective vaccine, although not a panacea, will be one of the key pioneers in helping to eliminate the threat from SARS-CoV-2 and controlling the COVID-19 pandemic. Although several vaccines are already under clinical trials with some being in advanced stages, it is also valuable to consider some other vaccines, which can prove to be instrumental in ultimately eliminating the virus. A Chinese-based company, Anhui Zhifei Longcom Biopharmaceutical, is designing a universal protein-subunit based vaccine, now in Phase 2 trials, which involves an artificial protein consisting of a spike receptor-binding domain (RBD) dimer, instead of the usual RBD monomer (91). While its design has been previously explored for other coronaviruses, its immunogenic activity has not been studied. A study published in the journal *Cell* showed that the RBD dimer design has the ability to generate a higher specific IgG response with a much more elevated NAb titer by up to 10–100-folds than a conventional RBD monomer vaccine, based on studies conducted on mice models (91). Another benefit to the RBD dimer design is that it has the ability to be manufactured on a large-scale which is ideal for a pandemic situation like COVID-19 (90). Although this novel approach is still very much at the early stage of development it will be interesting to closely monitor progress in the development of this vaccine platform.

Although several organizations including WHO have committed to ensuring that developing countries receive an adequate supply of vaccines, it is yet to be seen how politics will influence the outcome and which vaccine will ultimately be successful. Ideally a vaccine has to be made available to close to 8 billion people. Unanswered questions are: 1. Who will pay for the vaccine? Companies who manufacture the vaccines will require recovery of investment and cost of production. 2. How will the vaccine be distributed globally and how quickly? Global cooperation is required; however, most likely the initial distribution will be to the country (or countries) who produced the vaccine. Furthermore, while many are placing their hopes on a vaccine, it is also important to consider alternatives to a vaccine, should a vaccine be less effective than hoped or rapidly become ineffective. Ideally, the availability of effective drugs to protect against infection and reduce morbidity and mortality may also become available but at present there are only limited options (34). Thus, the continued implementation of social distancing and proper hygiene practices including the global availability of appropriate personal protective equipment will be essential. Since it is unclear how promptly global demand for a vaccine will be met, it is likely these other methods will need to be utilized for some time until vaccines are available for everyone. In the absence of a truly effective drug to prevent and/or treat COVID-19 we will likely need to follow the social distancing and hygiene precautions for the foreseeable future and, if necessary, rigorously enforce them so as to control the pandemic.

Even though the more vulnerable group of the population, such as the elderly, immunocompromised, and those with co-morbidities, will be given priority for vaccines, they are not usually included in clinical trials and thus the effectiveness of the vaccine and risk of side effects will be unknown in this population.

There are several vaccine candidates currently in clinical trials with AstraZeneca/Oxford's AZD1222, Moderna's mRNA1273 and Sinovac's CoronaVac vaccines advancing to Phase 3 clinical trials. With many placing their hopes on a vaccine against COVID-19 being available by the end of 2020 or early 2021, it is yet to be seen how the vaccine will be distributed, how national interests will unfold and whether the vaccine will ultimately prove to be safe and effective when administered to the global population at large.

## Author Contributions

OS reviewed the literature and prepared drafts of the manuscript for final submission. AS assisted in evaluation of the literature and edited drafts of the manuscript. HD assisted in evaluation of literature and finalizing manuscript for submission. CT initiated the review, provided input throughout, and finalized manuscript for submission. All authors contributed to the article and approved the submitted version.

## Conflict of Interest

The authors declare that the research was conducted in the absence of any commercial or financial relationships that could be construed as a potential conflict of interest.
